# *Gnathia bermudensis* (Crustacea, Isopoda, Gnathiidae), a new species from the mesophotic reefs of Bermuda, with a key to *Gnathia* from the Greater Caribbean biogeographic region

**DOI:** 10.3897/zookeys.891.39564

**Published:** 2019-11-21

**Authors:** Kerry A. Hadfield, Nikolaos V. Schizas, Tapas Chatterjee, Nico J. Smit

**Affiliations:** 1 Water Research Group, Unit for Environmental Sciences and Management, North-West University, Private Bag X6001, Potchefstroom, 2520, South Africa North-West University Potchefstroom South Africa; 2 University of Puerto Rico at Mayagüez, Department of Marine Sciences, PO Box 9000, Mayagüez, Puerto Rico, 00681, USA University of Puerto Rico at Mayagüez Mayagüez Puerto Rico; 3 Crescent International School, Bario, Govindpur, Dhanbad 828109, Jharkhand, India Crescent International School Dhanbad India

**Keywords:** Atlantic Ocean, benthic, ectoparasite, Nekton Mission, taxonomy

## Abstract

*Gnathia bermudensis***sp. nov.** is described from mesophotic coral ecosystems in Bermuda; it is distinguished by pronounced and pointed supraocular lobes, two superior frontolateral processes and a weak bifid mediofrontal process, pereonite 1 not fused dorsally with the cephalosome, and large eyes. This is the first record of a species of *Gnathia* from Bermuda. A synopsis and key to the other *Gnathia* species from the Greater Caribbean biogeographic region is provided.

## Introduction

Gnathiid isopods are temporary ectoparasites that occur in a variety of habitats ranging in depth, water currents, temperature, climate and salinity (Smit and Davies 2004). The parasitic juveniles feed on the blood and lymph of their fish hosts, while the non-feeding free-living adults are usually hidden in cavities, corals, or sponges (Hadfield et al. 2009). The taxonomic classification of these isopods is based almost exclusively on the morphology of the adult males, and this makes studies reliant on accurate species identification problematic as males can be difficult to obtain. Currently, there are 12 genera in the family Gnathiidae Leach, 1814 (Smit et al. 2019). Of these, the most speciose genus is *Gnathia* Leach, 1814, with 126 valid species (Boyko et al. 2008 onwards). To date, there are 14 known species of *Gnathia* from the Greater Caribbean biogeographic region (see Table 1 for a summary of known information on these species). In 1993, Müller (1993) proposed *Gnathia puertoricensis* Menzies & Glynn, 1968 as a junior synonym for *G. virginalis* Monod, 1926 based on the variation in the characters that separated these two species (granulation and tubercles on the anterior pereonites and cephalon). Although not recognised in subsequent publications on gnathiids from this region (George 2003; Farquharson et al. 2012), this synonymisation appears to still be valid and the information regarding both species is combined in Table 1.

**Table 1.** Summary of the location, depth, size and references of 15 *Gnathia* species from the Greater Caribbean biogeographic region, including the 14 previously known species and the new species, *Gnathia bermudensis* sp. nov.

Species	Location	Depth (m)	Size (mm)	Substratum	References
*G. beethoveni* Paul & Menzies, 1971	Venezuela	95	3	mangrove roots; muddy and sandy bottoms; algae; seaweed; tunicates; seagrass	Paul and Menzies 1971; Díaz et al. 2013
	Colombia (Santa Marta)	13–30		coral rubble	Müller 1988a
	Tobago				Kensley and Schotte 1994
	Mexico (Puerto Morelos)	3–12	1.8	coral rubble	Monroy-Velázquez and Alvarez 2016; Monroy-Velázquez et al. 2017
*G. bermudensis* sp. nov.	Bermuda	56–90	1.7–2.2	loose gravel and sediment (associated with corals); algae; sponges; rodoliths	Present study
*G. brucei* George, 2003	USA (North Carolina)	1000–1020	2.8–3.2		George 2003
*G. calsi* Müller, 1993	Martinique, French Antilles	0–2	1.9	dead corals	Müller 1993
*G. gonzalezi* Müller, 1988	Colombia (Santa Marta)	12–30	1.5	coral rubble	Müller 1988a
*G. hemingwayi* Ortiz & Lalana, 1997	Cuba (Cojímar Bay)	2	3	wood pile	Ortiz and Lalana 1997
*G. johanna* Monod, 1926	US Virgin Islands (St. John)	29–46	2–2.16		Monod 1926; Müller 1988b
	Colombia				Kensley and Schotte 1990
	Venezuela			seagrass beds; muddy bottom	Díaz et al. 2013
*G. magdalenensis* Müller, 1988	Colombia (Santa Marta)	6–30	2.8	coral rubble	Müller 1988a
	Belize				Kensley and Schotte 1989
	Mexico (Puerto Morelos)	3–12		coral rubble	Monroy-Velázquez et al. 2017
*G. marleyi* Farquharson, Smit & Sikkel, 2012	St. John, US Virgin Islands; Bahamas; British Virgin Islands (Guana Island); Puerto Rico; Saba (Lesser Antilles)	3–5	2.6–3.7	several host fish	Farquharson et al. 2012
*G. micheli* Ortiz, Winfield & Varela, 2012	Cuba (Cayo Matias)	20	2.6–3.3	algae	Ortiz et al. 2012
*G. rathi* Kensley, 1984	Belize (Carrie Bow Cay)	0.5–128	1.6–1.9	rubble	Kensley 1984
*G. samariensis* Müller, 1988	Colombia (Santa Marta)	30	1.7	coral rubble	Müller 1988a
*G. triospathiona* Boone, 1918	USA (Florida)	200	8.8		Boone 1918
*G. vellosa* Müller, 1988	Colombia (Santa Marta)	25–30	1.5	sponges and hydroids	Müller 1988a
	Venezuela			seagrass beds; mangrove roots; algae	Díaz et al. 2013
	Mexico (Puerto Morelos)	6–12	2.7	coral rubble	Monroy-Velázquez and Alvarez 2016; Monroy-Velázquez et al. 2017
*G. virginalis* Monod,1926	US Virgin Islands	29	2.2		Monod 1926
Syn: *G. puertoricensis* Menzies & Glynn, 1968	Puerto Rico	0–3	3		Menzies and Glynn 1968
	Cuba				Ortiz 1983; Müller 1988a
	Belize (Carrie Bow Cay)			rubble	Kensley 1984
	Colombia (Santa Marta)	0–30	2	coral rubble; under stones; fouling on harbour pilings	Müller 1988a
	Martinique, French Antilles	0.5–2		seagrass beds; dead corals; under stones	Müller 1993
	Venezuela			mangrove roots; seagrass beds; muddy bottom; algae	Díaz et al. 2013
	Mexico (Puerto Morelos)	6–12	2.2	coral rubble	Monroy-Velázquez and Alvarez 2016; Monroy-Velázquez et al. 2017

Recently, there has been a growing interest in gnathiids from this region specifically regarding their role in cleaner interactions (Artim et al. 2017), food web ecology (Demopoulos and Sikkel 2015), and their role as potential vectors of blood parasites (Cook et al. 2015). However, all of this work has focused on a single species, *G. marleyi* Farquharson, Smit & Sikkel, 2012, and therefore it is also the only species from this region with known hosts for the parasitic larval stage. These host fishes include *Acanthurus bahianus* Castelnau, 1855; *Chaetodon capistratus* Linnaeus, 1758; *Epinephelus guttatus* (Linnaeus, 1758); *Haemulon flaviolineatum* (Desmarest, 1823); *H. plumieri* (Lacepede, 1801); *H. sciurus* (Shaw, 1803); *Holocentrus rufus* (Walbaum, 1792); *Lutjanus apodus* (Walbaum, 1792); *L. griseus* (Linnaeus, 1758); *Scarus taeniopterus* Desmarest, 1831; *Sparisoma aurofrenatum* (Valenciennes, 1840); *Stegastes diencaeus* (Jordan & Rutter, 1897); and *S. planifrons* (Cuvier, 1830) (see Farquharson et al. 2012).

Bermuda forms part of this Greater Caribbean biogeographic region in the North Atlantic Ocean (Robertson and Cramer 2014). It is situated on the western side of the Sargasso Sea (high salinity, high temperatures and high biodiversity), and has the most northern coral reef system in the world. As part of the Nekton Foundation/XL-Catlin Deep-Ocean Survey – Mission 1 (www.nektonmission.org), fish (Stefanoudis et al. 2019a), zooplankton (Stefanoudis et al. 2019b), black corals (Wagner and Shuler 2017), macroalgae (Schneider et al. 2018, 2019) and other benthic communities (NVS pers. obs.) were studied. Macrofaunal collections from mesophotic reef ecosystems of Bermuda (MCEs) contained several specimens of a gnathiid isopod that did not correspond to currently described species. This isopod is here described as a new species of *Gnathia* and is the first gnathiid isopod to be recorded from Bermuda.

## Materials and methods

**Figure 1.**
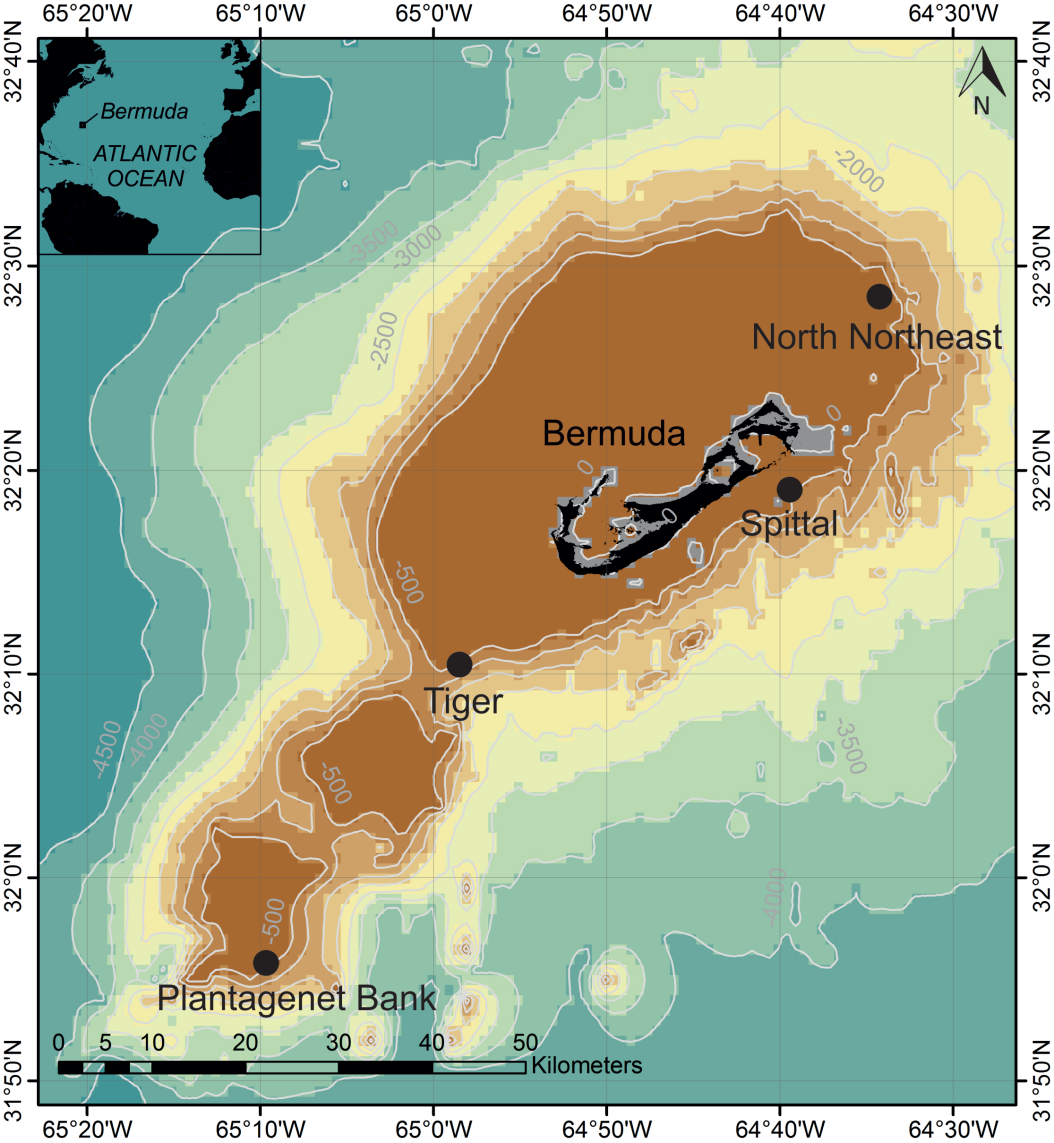
Map of collection sites around Bermuda. Data overlay GEBCO_2014 Grid which provides 30 arc-second global grid of elevations. Depth contours in meters.

All benthic samples were collected from 17 July to 14 August 2016 aboard the R/V “Baseline Explorer”. Mesophotic benthic surveys and sampling were conducted using Trimix rebreathing divers from the Global Underwater Explorers (**GUE**) down to 94 m around the edge of the Bermuda platform. The sampling sites North Northeast (**NNE**), Plantagenet Bank, Spittal, and Tiger, were selected along the northeast, southeast and southern slopes of the Bermuda platform, respectively (Figure 1). During the same mission, two two-person Triton Class Submersibles (Nomad and Nemo; Vero Beach, FL, United States) equipped with an arm manipulator assisted in sample collection down to 300 m. Divers collected macroalgae, loose gravel, bottom sediment, rhodoliths, sponges, and hard and soft corals to characterise the biodiversity of the Bermudian mesophotic reefs. The depth range for each sample was noted. Once the substrata were brought onto the research vessel, they were placed on a 0.063 μm sieve and washed thoroughly with filtered water. Meiofauna and macrofauna associated with the substrata were captured on the 0.063 μm sieve and preserved in > 95 % ethanol. The preserved samples were sorted, placed in 95 % ethanol, and stored at -20 °C until further processing. Research permits for Bermuda were issued by the Department of Environment and Natural Resources, Bermuda (No. 2016070751).

From these samples, several gnathiids were cleaned and prepared for scanning electron microscopy (SEM; PhenomWorld). Gnathiids were also observed and drawn using an Olympus BX41 compound microscope and an Olympus SZX7 dissecting microscope with a camera lucida. Appendages were removed with the aid of dissecting needles and forceps and stained using lignin pink.

The species description was prepared in DELTA (DEscriptive Language for TAxonomy) using a general Gnathiidae character set (as used in Svavarsson and Bruce 2012). The description is based on the adult male gnathiid. Terminology follows Monod (1926), Cohen and Poore (1994) and Svavarsson and Bruce (2012, 2019). Isopod classification follows that of Brandt and Poore (2003).

Material is deposited in the Natural History Museum of Bermuda.

## Taxonomy

### Suborder Cymothoida Wägele, 1989

**Superfamily Cymothooidea Leach, 1814**

**Family Gnathiidae Leach, 1814**

#### Genus Gnathia

Taxon classificationAnimaliaIsopodaGnathiidae

Leach, 1814, restricted syn.

CE5D4785-98B5-5ED3-9DDA-3DECEB9CB043

Gnathia Leach, 1814: 386–402; Monod 1926: 326–329 (part); Cohen and Poore 1994: 343–346.
Anceus Risso, 1816: 8.
Praniza Latreille, 1817: 54.
Zuphea Risso, 1826: 104.
Gnathia (Gnathia) s.s.: Monod 1926: 329 (part).
Gnathia (Perignathia) : Monod 1926: 554–555 (not Perignathia Monod, 1922).

##### Type species.

*Gnathia termitoides* Leach, 1814, by monotypy (see Cohen and Poore 1994).

##### Diagnosis.

Frontal margin of cephalosome generally straight (not deeply excavated), with frontal processes. Mandibles not elongate, usually with mandibular incisor and dentate mandibular blade. Paraocular ornamentation and/or a dorsal sulcus may be present on cephalosome. Pereonite 1 possibly immersed in cephalosome. Pylopod broad and distinct, with two or three articles, operculate; article 1 enlarged, generally with dense external margin of plumose setae; article 3 reduced or absent.

##### Remarks.

*Gnathia* can be identified by the presence of frontal processes, a straight frontal border, a broad 2 or 3 articled pylopod, and non-extended mandibles with a dentate blade.

It is the most speciose genus in the family Gnathiidae (currently with 126 valid species). *Gnathia* is a cosmopolitan genus, commonly found in coral-reef habitats, and its parasitic larvae have been reported from both teleost and elasmobranch hosts (Smit and Davies 2004). The most recent revision of this genus was by Cohen and Poore (1994).

#### Gnathia bermudensis sp. nov.

Taxon classificationAnimaliaIsopodaGnathiidae

67D5CD94-2B3E-506B-B35D-7D69423287ED

http://zoobank.org/5FD1EC92-2EE5-40E8-8BB8-0C47255A73A2

Figures 2 , 3 , 4

##### Material examined.

***Holotype*.** Bermuda • 1 ♂ (2.2 mm TL); Plantagenet Bank (31°56.55'N, 65°09.29'W); 56 m; 12 Aug 2016; Diver 2, from sediment; Sample ID BEX 2016-449 (BAMZ 2016-338-147).

***Paratypes*.** Bermuda • 3 ♂♂ (1.9–2.1 mm TL) (one dissected), 1 ♂ used for SEM (1.8 mm TL), 1 ♀ (1.6 mm TL); same info as holotype (BAMZ 2016-338-148).

##### Other material.

Bermuda • 4 ♂♂ (1.8–1.9 mm TL) (one dissected); Spittal (32°19.119'N, 64°39.437'W); 45 m; 3 Aug 2016; sediment from *Montastraea cavernosa* (Linnaeus, 1767) corals, Divers 39; Sample ID BEX 2016-227, Parent BEX2016-225 (sediment from several *Montastraea cavernosa* colonies) (BAMZ 2016-338-149) • 1 ♂ (2.0 mm TL); NNE (32°28.59'N, 64°34.46'W); 90 m; 4 Aug 2016; Event Divers; Sample ID BEX 2016-250, Parent BEX2016-248 (BAMZ 2016-338-150) • 1 zuphea (Z1) (0.45 mm TL); NNE (32°28.59'N, 64°34.46'W); 4 Aug 2016; algae substrate; Sample ID BEX 2016-251 • 1 ♂ used for SEM (1.7 mm TL); Spittal (32°19.119'N, 64°39.437'W); from rhodolith collected between 82–152 m; 7 Aug 2016; Dive 22, Nomad 1 (a Triton Submersible); Sample ID BEX 2016-299, Parent BEX2016-0265 • 1 ♂ (2.0 mm TL), 1 ♀ (1.9 mm TL), 1 zuphea (0.8 mm TL); Tiger 4 (32°11.17'N, 64°58.36'W); 7 Aug 2016; Divers 12, from sediment; Sample ID BEX 2016-304, Parent BEX2016-0282 (rhodolith with red encrusting sponge, > 40 m) (BAMZ 2016-338-151) • 2 ♂♂ (1.9–2.0 mm TL); Spittal (32°19.119'N, 64°39.437'W); 77 m; 11 Aug 2016; wash from rhodolith; Sample ID BEX 2016-428 • 1 ♂ (2.0 mm TL), 1 praniza (P3) (2.3 mm TL), 1 zuphea (Z1) (0.5 mm TL); Spittal (32°19.119'N, 64°39.437'W); 77 m; 11 Aug 2016; Diver 30; Sample ID BEX 2016-430 • 4 zuphea (Z1) (0.5 mm TL); Plantagenet Bank (31°56.55'N, 65°09.29'W); 56 m; 12 Aug 2016; Divers 2; Sample ID BEX 2016-450 • 2 ♂♂ (1.7–1.9 mm TL) (one used for SEM); Plantagenet Bank (31°56.55'N, 65°09.29'W); 56 m; 12 Aug 2016; Divers 6; Sample ID BEX 2016-451. All samples were collected by GUE technical divers except Sample ID BEX 2016-299, Parent BEX2016-0265, which was collected by a Triton Submersible.

**Figure 2.**
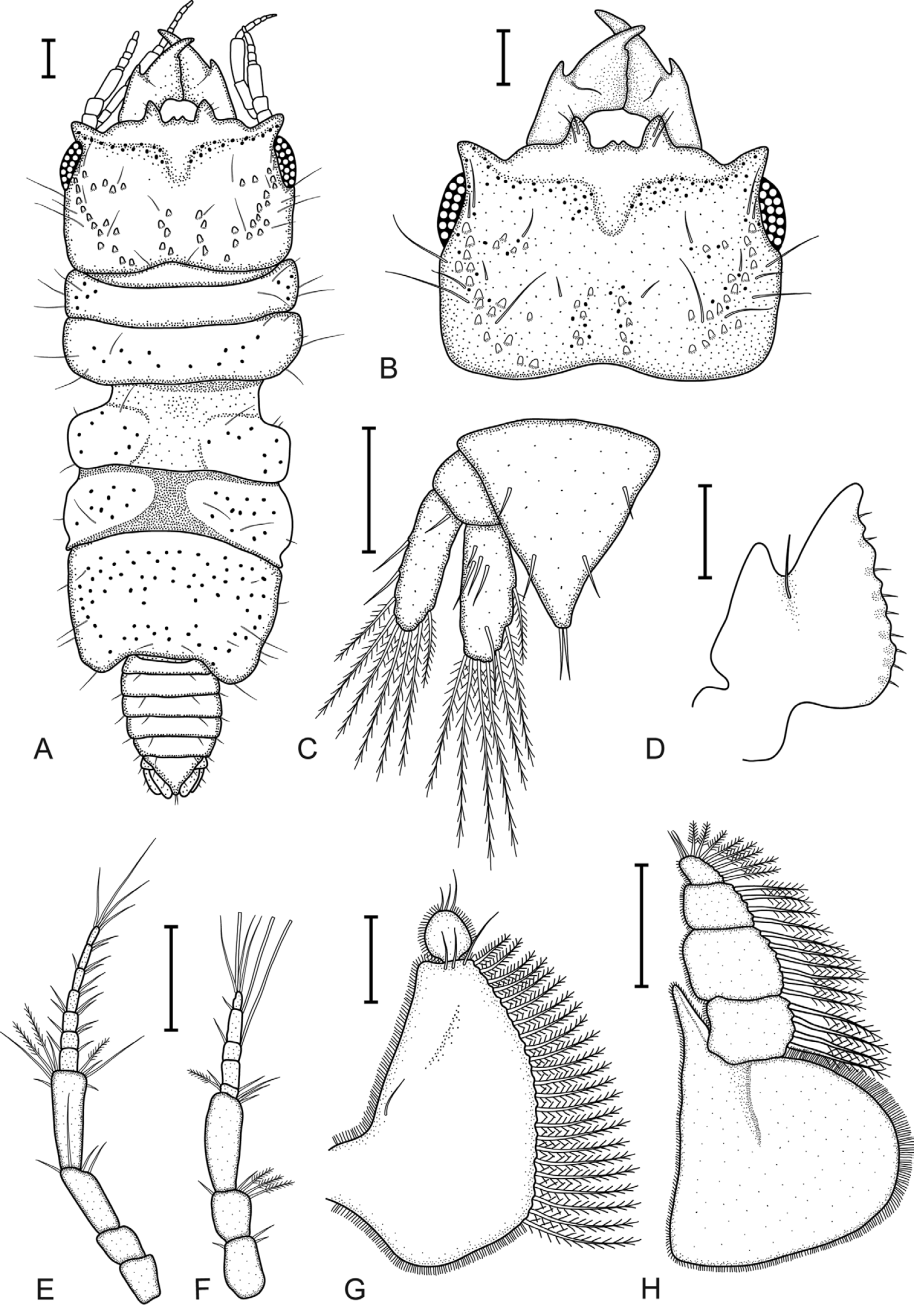
*Gnathia bermudensis* sp. nov. (BAMZ 2016-338-147), male holotype (2.2 mm TL) **A** dorsal view **B** dorsal view of cephalosome **C** dorsal view of pleotelson and uropods **D** dorsal view of mandible **E** antenna **F** antennula **G** pylopod **H** maxilliped. Scale bars: 100 μm.

##### Description of male.

*Body* 2.3 times as long as greatest width, widest at pereonite 3; dorsal surfaces sparsely punctate, sparsely setose. *Cephalosome* quadrate, 0.7 as long as wide, lateral margins sub-parallel; dorsal surface with sparse granules; dorsal sulcus narrow, shallow, short; translucent region absent; paraocular ornamentation strongly developed, posteromedian tubercle present. *Frontolateral* present. *Frontal margin* slightly produced. *External scissura* present, wide, shallow. *process* present, weak, bifid, without fine setae. *Supraocular lobe* pronounced, pointed; accessory supraocular lobe not pronounced. *Superior frontolateral process* present, single, strong, conical, with two long simple setae. *Inferior frontolateral process* absent. *Mesioventral margin* concave. *Eyes* present, elongate, 0.3 times as long as cephalosome length, bulbous, standing out from head surface, ommatidia arranged in rows, eye colour black.

*Pereon* lateral margins subparallel, with few setae; anteriorly with sparse fine granules. *Pereonite 1* not fused dorsally with cephalosome; dorsolateral margins fully obscured by cephalosome. *Pereonite 2* wider than pereonite 1. *Areae laterales* present on pereonite 5. *Pereonite 6* without lobi laterales; lobuii weak, globular. *Pleon* covered in pectinate scales, epimera not dorsally visible on all pleonites. *Pleonite 1* lateral margins with one pair of simple setae, with one pair of simple setae medially. *Pleotelson* as long as anterior width, covered in pectinate scales. Pleotelson lateral margins finely serrate, anterolateral margins weakly convex, with two submarginal setae; posterolateral margin distally weakly concave, with two submarginal setae; apex with two setae.

**Figure 3.**
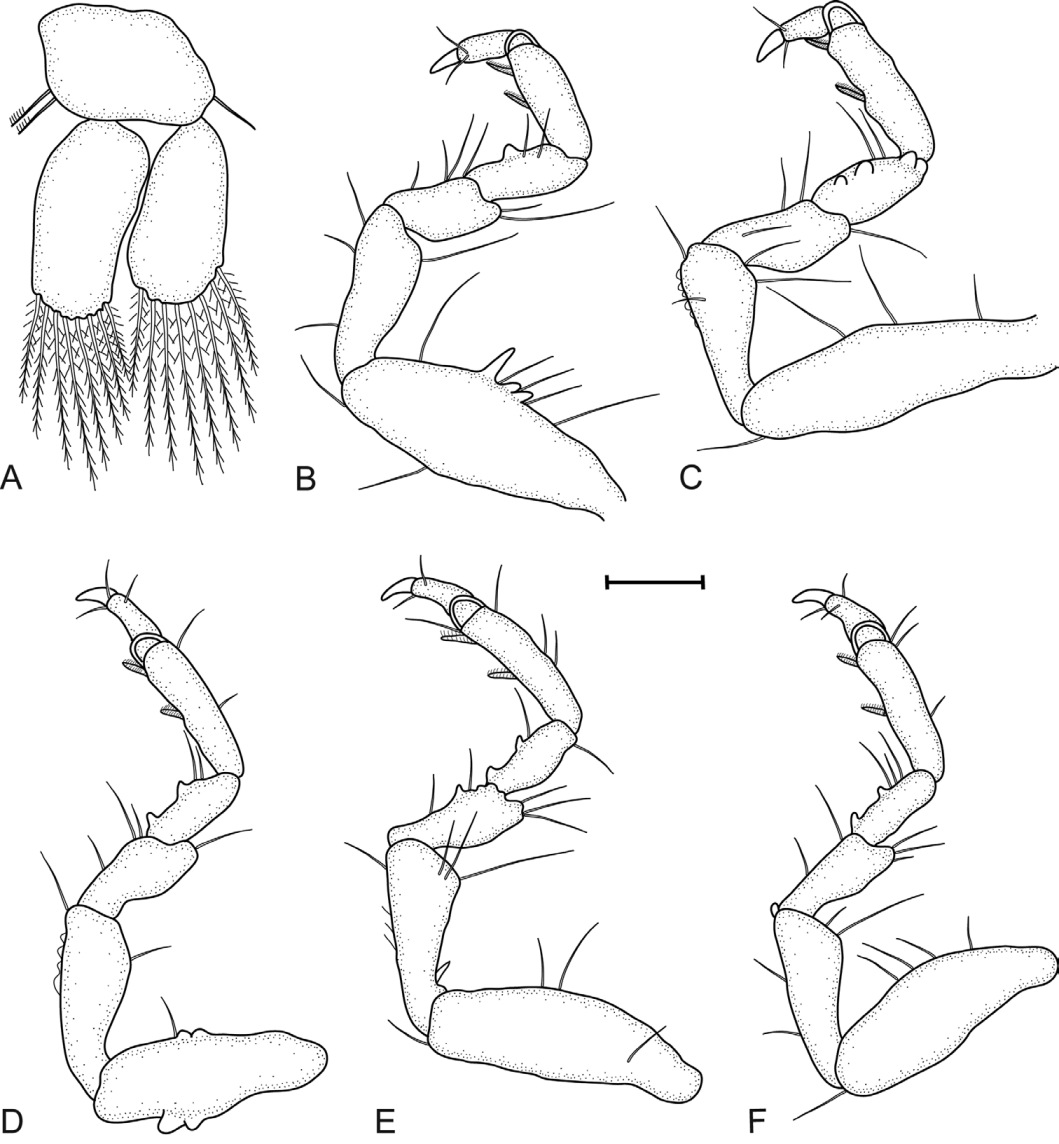
*Gnathia bermudensis* sp. nov. (BAMZ 2016-338-147), male holotype (2.2 mm TL) **A** pleopod 2 **B–F** pereopods 2–6, respectively. Scale bar: 100 μm.

*Antennula* peduncle article 2 0.8 times as long as article 1; article 3 1.9 times as long as article 2, 2.7 times as long as wide; flagellum 1.1 times as long as article 3, with five articles; article 3 with one aesthetasc seta and one simple seta; article 4 with one aesthetasc seta and one simple seta; article 5 terminating with one aesthetasc seta and three simple setae. *Antenna* peduncle article 4 2.5 times as long as wide, twice as long as article 3, and four simple setae; article 5 1.3 times as long as article 4, 2.8 times as long as wide, inferior margin with three penicillate setae, with six simple setae; flagellum 1.5 times as long as article 5, with seven articles.

*Mandible* 0.4 as long as width of cephalosome, triangular, weakly curved, evenly; apex 42% total length; mandibular seta present. *Incisor* dentate. *Blade* present, dentate, weakly convex, dentate along 100% of margin. *Pseudoblade* absent; internal lobe absent; dorsal lobe absent; basal neck short; erisma present.

*Maxilliped* 5-articled; article 1 lateral margin with continuous marginal scale-setae; article 2 lateral margin with four plumose setae; article 3 lateral margin with six plumose setae; article 4 lateral margin with four plumose setae; article 5 with eight plumose setae; endite extending to mid-margin of article 3; without coupling setae.

**Figure 4.**
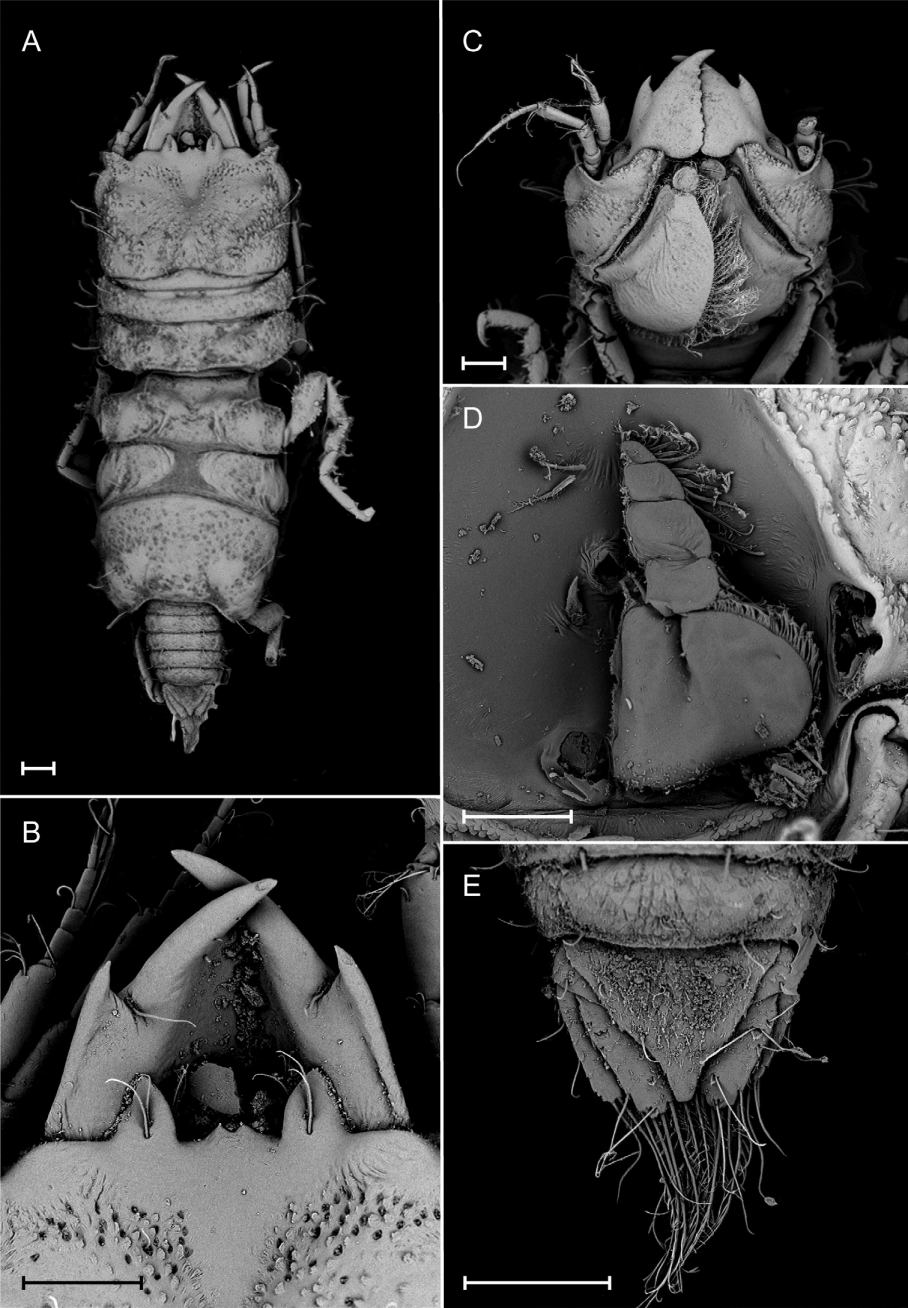
*Gnathia bermudensis* sp. nov. (BAMZ 2016-338-148), male paratype (1.8 mm TL) Scanning Electron Microscope (SEM) images. **A** dorsal view **B** frontal margin and mandibles **C** ventral view of cephalosome **D** maxilliped **E** dorsal view of pleotelson and uropods. Scale bars: 100 μm.

*Pylopod* first article 1.5 as long as wide, without distolateral lobe; posterior and lateral margins forming rounded curve; lateral margin with 23 large plumose setae; mesial margin with continuous scale-setae; distal margin with three simple setae; second article 1.1 as long as wide.

*Pereopods 2–6* with long simple setae and randomly covered in pectinate scales; pereopod 2 with tubercles on carpus and basis to ischium. *Pereopod 2 basis* 2.8 times as long as greatest width, superior margin with five setae, inferior margin with two setae; ischium 0.6 times as long as basis, 2.6 as long as wide, superior margin with one seta, inferior margin with three setae; merus 0.5 as long as ischium, 1.5 as long as wide, superior margin with two setae, inferior margin with four setae; carpus 0.6 as long as ischium, 1.9 as long as wide, superior margin without setae, inferior margin with two setae; propodus 0.8 times as long as ischium, 2.8 times as long as wide, superior and inferior margins without setae, and two robust setae; dactylus 0.7 as long as propodus. *Pereopods 3 and 4* similar to pereopod 2. *Pereopod 5* similar to pereopod 6. *Pereopod 6* with tubercles on merus and carpus; basis 3.1 times as long as greatest width, superior margin with two setae, inferior margin with two setae; ischium 0.7 as long as basis, 2.7 as long as greatest width, superior margin with three setae, inferior margin with four setae; merus 0.6 as long as ischium, 2.1 times as long as wide, superior margin with three setae, inferior margin with two setae; carpus 0.6 as long as ischium, 1.7 times as long as wide, superior margin and inferior margin with one seta; propodus 0.9 as long as ischium, 3.8 times as long as wide, superior margin with three setae, inferior margin with one seta, and two robust setae; dactylus 0.6 as long as propodus.

*Penes* opening flush with surface of sternite 7.

*Pleopod 2 exopod* 1.9 as long as wide, distally broadly rounded, with eight plumose setae; endopod 1.9 as long as wide, distally broadly rounded, with eight plumose setae; appendix masculina absent; peduncle 1.5 times as wide as long, mesial margin with two coupling setae, lateral margin with one simple seta.

*Uropod* rami extending beyond pleotelson, apices narrowly rounded. *Uropod endopod* 2.4 as long as greatest width, dorsally with five setae; lateral margin straight; proximomesial margin weakly convex, with seven long plumose setae. *Uropod exopod* not extending to end of endopod, 2.9 times as long as greatest width; lateral margin straight, with two simple setae; proximomesial margin straight, distally convex, mesiodistal margin with seven long plumose setae.

##### Etymology.

The epithet *bermudensis* is for the country Bermuda, being the first *Gnathia* record from this island nation.

##### Distribution.

Bermuda.

##### Hosts.

Not known.

##### Remarks.

*Gnathia bermudensis* sp. nov. may be identified by the produced frontal margin; presence of two superior frontolateral processes; a weak and bifid mediofrontal process; and pronounced and pointed supraocular lobes. The uropod rami extend past the posterior point of the pleotelson; pereonite 1 is not dorsally fused with the cephalosome; large eyes (0.3 as long as cephalosome length); and a weakly curved, dentate mandible.

This species is from a moderate depth of 56–90 m and was collected from several habitat types (algae, loose gravel, rhodoliths, sediment associated with scleractinian corals, muddy sand, and sponges) encompassing the mesophotic reef ecosystems of Bermuda. The Mesophotic Coral Ecosystems (MCEs) of Bermuda represent the most northern coral reef systems of the Atlantic; they are visually dominated by scleractinian corals at the upper depth limits, which are replaced gradually at greater depths by rhodoliths, macroalgae beds and fossilised reefs (Goodbody-Gringley et al. 2019). The new gnathiid species has been found on the mesophotic slopes of the main seamount (i.e., the main island of Bermuda) and the smaller seamount Plantagenet (Figure 1); therefore, it is expected to be found throughout the deeper reefs of Bermuda. Only four other species of *Gnathia* have been collected from greater depths in this region.

*Gnathia bermudensis* sp. nov. is most similar to *G. beethoveni* Paul & Menzies, 1971, *G. calsi* Müller, 1993, *G. johanna* Monod, 1926, *G. magdalenensis* Müller, 1988, and *G. virginalis* Monod, 1926 from the region. The frontal margin of *G. beethoveni* differs from *Gnathia bermudensis* in having less pronounced supraocular lobes, four frontolateral processes, a shallow median notch, and the cephalosome is lacking dorsal tubercles. *Gnathia calsi* also has a deeply notched mediofrontal process with two lobes (and setae), and well developed but angular supraocular lobes, not seen in *Gnathia bermudensis* sp. nov. *Gnathia johanna* is narrower than *Gnathia bermudensis* sp. nov., with less pronounced supraocular lobes and a single convex mediofrontal process (with setae) between the superior frontolateral processes. *Gnathia magdalenensis* and *G. virginalis* differ from *Gnathia bermudensis* sp. nov. in having slightly pointed supraocular lobes, a single pointed mediofrontal process with setae, and a longer cephalosome that is fused with pereonite 1.

Although adult females and zuphea juveniles were collected with the males, they cannot be confidently linked to this species without molecular or ecological data. More collections and rearing of the gnathiid isopods would need to be made in the future for more information and validation of these different life stages, as well as to determine the hosts of the juvenile stages.

### Key to members of the genus *Gnathia* known from the Greater Caribbean biogeographic region

This key is based on the morphological characters of the adult male:

**Table:** 

1	Pereonite 5 elongate (quadrate); located in deeper waters (≥ 200 m); cephalon frontal border wavy (with 3 bifid frontal lobes or 3 tooth-like projections)	**2**
–	Pereonite 5 similar in shape and size to pereonites 2–4; located in shallower waters (≤ 200 m); cephalon frontal border with regular frontal processes	**3**
2	Frontal border produced with large quadrate projection; deep sea (> 1000 m); total body length measuring approximately 2.8–3.2 mm	***G. brucei***
–	Frontal border with deep V-shaped grove; depths below 1000 m (approx. 200 m); total body length measuring approximately 8.8 mm	***G. triospathiona***
3	Mediofrontal processes absent	**4**
–	Mediofrontal processes present	**10**
4	Anterior margin of cephalon medially concave; robust body; cephalon wider than long and without granules or tubercles	***G. gonzalezi***
–	Anterior margin of cephalon not medially concave; slender body; cephalon quadrate	**5**
5	Only superior frontolateral processes present	**6**
–	Both superior and inferior frontolateral processes present	**7**
6	Frontal margin slightly convex or straight; cephalon granular (tubercles)	***G. rathi***
–	Frontal margin convex with 4 medial setae; cephalon without tubercles	***G. johanna***
7	Pylopod 2-articled; inferior frontolateral processes smaller in size than superior frontolateral processes	***G. micheli***
–	Pylopod 3-articled; superior and inferior frontolateral processes similar in size	**8**
8	Cephalon and body without granules or tubercles; sparsely setose	***G. beethoveni***
–	Cephalon with granules or tubercles; few to many slender setae over the body	**9**
9	Supraocular lobes not well developed; narrow pleon and pleotelson longer than wide; pereonites 5 and 6 not clearly defined	***G. hemingwayi***
–	Supraocular lobes well developed; pleon with short setae and wider than long; pereonites 5 and 6 clearly defined	***G. calsi***
10	Mediofrontal process bifid	**11**
–	Mediofrontal process not bifid	**12**
11	Frontal margin medially concave; superior frontolateral processes weak with 3 or 4 simple setae on each process; supraocular lobe not pronounced	***G. marleyi***
–	Frontal margin produced; superior frontolateral processes strong with 2 simple setae on each process; supraocular lobe pronounced and pointed	***G. bermudensis* sp. nov.**
12	Cephalon with few or no granules or tubercles	**13**
–	Cephalon with many small tubercles (finely granular)	**14**
13	Mediofrontal process with 2–4 simple setae; mandible with inner lobe	***G. magdalenensis***
–	Mediofrontal process without any setae; mandible without inner lobe	***G. samariensis***
14	Cephalon approximately 1.7 times as wide as long; mandibular carina distally notched	***G. vellosa***
–	Cephalon approximately 1.2 times as wide as long; mandibular carina distally rounded	***G. virginalis***

## Supplementary Material

XML Treatment for Gnathia

XML Treatment for Gnathia bermudensis

## References

[B1] Artim JM Hook A Grippo RS Sikkel PC (2017) Predation on parasitic gnathiid isopods on coral reefs: a comparison of Caribbean cleaning gobies with non-cleaning microcarnivores. Coral Reefs 36: 1213–1223. 10.1007/s00338-017-1613-6

[B2] Boone PL (1918) Description of ten new isopods. Proceedings of the United States National Museum 54: 591–604.

[B3] Brandt A Poore GCB (2003) Higher classification of the flabelliferan and related Isopoda based on a reappraisal of relationships. Invertebrate Systematics 17: 893–923. 10.1071/IS02032

[B4] Boyko CB, Bruce NL, Hadfield KA, Merrin KL, Ota Y, Poore GCB, Taiti S, Schotte M, Wilson GDF (Eds) (2008 onwards) World Marine, Freshwater and Terrestrial Isopod Crustaceans database. *Gnathia* Leach, 1814. Accessed through: World Register of Marine Species. http://www.marinespecies.org/aphia.php?p=taxdetails&id=118437 [on 2019-07-10]

[B5] Cohen BF Poore GCB (1994) Phylogeny and biogeography of the Gnathiidae (Crustacea: Isopoda) with descriptions of new genera and species, most from South-Eastern Australia. Memoirs of the Museum of Victoria 54: 271–397. 10.24199/j.mmv.1994.54.13

[B6] Cook CA Sikkel PC Renoux LP Smit NJ (2015) Blood parasite biodiversity of reef-associated fishes of the eastern Caribbean. Marine Ecology Progress Series 533: 1–13. 10.3354/meps11430

[B7] Demopoulos AWJ Sikkel PC (2015) Enhanced understanding of ectoparasite-host trophic linkages on coral reefs through stable isotope analysis. International Journal for Parasitology: Parasites and Wildlife 4: 125–134. 10.1016/j.ijppaw.2015.01.002 25830112 PMC4356874

[B8] Díaz YJ Martín A Herrera J (2013) Diversidad de isópodos (Crustacea: Isopoda) del Parque Nacional Morrocoy, Venezuela, y clave de identificación. Boletín del Instituto Oceanográfico de Venezuela 52: 33–60.

[B9] Farquharson C Smit NJ Sikkel PC (2012) *Gnathia marleyi* sp. nov. (Crustacea, Isopoda, Gnathiidae) from the Eastern Caribbean. Zootaxa 3381: 47–61. 10.11646/zootaxa.3381.1.3

[B10] George RY (2003) Two new species of gnathiid isopod Crustacea from the North Carolina coast. Journal of the North Carolina Academy of Science 119: 33–40.

[B11] Goodbody-Gringley G Noyes T Smith SR (2019) Mesophotic Coral Ecosystems of Bermuda. In: Loya Y Puglise KA Bridge TCL (Eds) Mesophotic Coral Ecosystems (MCEs).Springer International Publishing, 31–45. 10.1007/978-3-319-92735-0

[B12] Hadfield KA Smit NJ Avenant-Oldewage (2009) Life cycle of the temporary fish parasite, *Gnathia pilosus* (Crustacea: Isopoda: Gnathiidae) from the east coast of South Africa. Journal of the Marine Biological Association of the United Kingdom 89: 1331–1339. 10.1017/S0025315409000587

[B13] Kensley B (1984) The Atlantic Barrier Reef Ecosystem at Carrie Bow Cay, Belize, III: New marine Isopoda. Smithsonian Institution Press, Washington, DC, 81 pp 10.5479/si.01960768.24.1

[B14] Kensley B Schotte M (1989) Guide to the marine isopod crustaceans of the Caribbean. Smithsonian Institution Press, Washington D.C., 308 pp 10.5962/bhl.title.10375

[B15] Kensley B Schotte M (1994) Marine isopods from the Lesser Antilles and Colombia (Crustacea: Peracarida). Proceedings of the Biological Society of Washington 107: 482–510.

[B16] Latreille PA (1817) Les Crustacés, les Arachnides, et les Insectes. In: Cuvier G (Ed.) Le Règne Animal, distribué d’après son organisation, pour servir de base à l’histoire naturelle des animaux et d’introduction à l’anatomie comparée. Vol. 3. D’Eterville, Paris, 653 pp 10.5962/bhl.title.41460

[B17] Leach WE (1814) Crustaceology. In: Brewster’s Edinburgh Encyclopedia. 7: 383–437. 10.5962/bhl.title.30911

[B18] Menzies RJ Glynn PW (1968) The common marine isopod Crustacea of Puerto Rico: A handbook for marine biologists. Martinus Nijhoff, The Hague, Netherlands, 133 pp.

[B19] Monod T (1926) Les Gnathiidæ. Essai monographique (morphologie, biologie, systématique). Mémoires de la Société des Sciences Naturelles du Maroc 13: 1–668.

[B20] Monroy-Velázquez V Alvarez F (2016) New records of isopods (Crustacea: Peracarida: Isopoda) from the Mesoamerican Reef at Puerto Morelos, Quintana Roo, Mexico. Check List 12: 1938. 10.15560/12.4.1938

[B21] Monroy-Velázquez V Rodríguez-Martínez RE Alvarez F (2017) Taxonomic richness and abundance of cryptic peracarid crustaceans in the Puerto Morelos Reef National Park, Mexico. PeerJ 5: e3411. 10.7717/peerj.3411 PMC5474090 28630800

[B22] Müller H-G (1988a) The genus *Gnathia* Leach (Isopoda) from the Santa Marta area, northern Colombia, with a review of Gnathiidea from the Caribbean Sea and Gulf of Mexico. Bijdragen tot de Dierkunde 58: 88–104. 10.1163/26660644-05801008

[B23] Müller H-G (1988b) Redescription of *Gnathia johanna* Monod, 1926 (Isopoda) from St. John, Virgin Islands. Bulletin Zoölogisch Museum Universiteit van Amsterdam 11(15): 129–135.

[B24] Müller H-G (1993) Marine Isopoda from Martinique, French Antilles: Cirolanidae and Gnathiidae (Crustacea: Cymothoidea). Cahiers de Biologie Marine 34: 29–42.

[B25] Ortiz M (1983) Guía para la identificación de los isópodos y tanaidáceos (Crustacea: Peracarida), asociados a los pilotes de las aguas Cubanas. Revista de Investigaciones Marinas 4: 3–20.

[B26] Ortiz M Lalana R (1997) *Gnathia hemingwayi* especie nueva (Isopoda, Gnathiidea) de la costa noroccidental de Cuba. Revista de Investigaciones Marinas 18: 21–26.

[B27] Ortiz M Winfield I Varela C (2012) First records of peracarid crustaceans from the Cayo Matias Ocean Blue Hole, SW Cuba, with the description of two new species. Zootaxa 3505: 53–66. 10.11646/zootaxa.3505.1.4

[B28] Paul AZ Menzies RJ (1971) Sub-tidal isopods of the Fosa de Cariaco, Venezuela, with descriptions of two new genera and twelve new species. Boletin de Instituto Universidade Oriente 10: 29–48.

[B29] Risso A (1816) Histoire naturelle des Crustacés des environs de Nice. Paris: Librairie Grecque-Latine-Allemande. 175 pp. 10.5962/bhl.title.8992

[B30] Risso A (1826) Histoire naturelle des principales productions de l’Europe méridionale et particulièrement de celles des environs de Nice et des Alpes Maritimes, vol. 5. FG Levrault, Paris, 403 pp 10.5962/bhl.title.58984

[B31] Robertson DR Cramer KL (2014) Defining and dividing the Greater Caribbean: Insights from the biogeography of shorefishes. PLoS ONE 9(7): e102918. 10.1371/journal.pone.0102918 PMC4108436 25054225

[B32] Schneider CW Lane CE Saunders GW (2018) A revision of the genus *Cryptonemia* (Halymeniaceae, Rhodophyta) in Bermuda, western Atlantic Ocean, including five new species and *C. bermudensis* (Collins & M. Howe) comb. nov. European Journal of Phycology 53: 350–368. 10.1080/09670262.2018.1452297

[B33] Schneider CW Popolizio TR Saunders GW (2019) Collections from the mesophotic zone off Bermuda reveal three species of Kallymeniaceae (Gigartinales, Rhodophyta) in genera with transoceanic distributions. Journal of Phycology 55: 414–424. 10.1111/jpy.12828 30565687

[B34] Smit NJ Davies AJ (2004) The curious life-style of the parasitic stages of gnathiid isopods. Advances in Parasitology 58: 289–391. 10.1016/S0065-308X(04)58005-3 15603765

[B35] Smit NJ Bruce NL Hadfield KA (2019) Parasitic Crustacea: State of knowledge and future trends. Springer International Publishing, 481 pp. 10.1007/978-3-030-17385-2

[B36] Stefanoudis PV Gress E Pitt JM Smith SR Kincaid T Rivers M Andradi-Brown DA Rowlands G Woodall LC Rogers AD (2019a) Depth-dependent structuring of reef fish assemblages from the shallows to the rariphotic zone. Frontiers in Marine Science 6 p. 357. 10.3389/fmars.2019.00307

[B37] Stefanoudis PV Rivers M Ford H Yashayaev IM Rogers AD Woodall LC (2019b) Changes in zooplankton communities from epipelagic to lower mesopelagic waters. Marine Environmental Research 146: 1–11. 10.1016/j.marenvres.2019.02.014 30879698

[B38] Svavarsson J Bruce NL (2012) New and little-known gnathiid isopod crustaceans (Cymothoida) from the northern Great Barrier Reef and the Coral Sea. Zootaxa 3380: 1–33. 10.11646/zootaxa.3380.1.1 31717118

[B39] Svavarsson J Bruce NL (2019) New gnathiid isopod crustaceans (Cymothoida) from Heron Island and Wistari Reef, southern Great Barrier Reef. Zootaxa 4609: 31–67. 10.11646/zootaxa.4609.1.2 31717118

[B40] Wagner D Shuler A (2017) The black coral fauna (Cnidaria: Antipatharia) of Bermuda with new records. Zootaxa 4344: 367–379. 10.11646/zootaxa.4344.2.11 29245639

